# Efficacy of Variable Doses of Prophylactic Intravenous Ondansetron in Attenuating Spinal Induced Hypotension in Parturients Undergoing Caesarean Delivery: A Randomised Control Trial

**DOI:** 10.7759/cureus.29440

**Published:** 2022-09-22

**Authors:** Sumedha Vashishth, Jatin Lal, Nidhi Bangarwa, Jitendra Wadhwani, Manu Smriti

**Affiliations:** 1 Anaesthesiology and Critical Care, Pandit Bhagwat Dayal Sharma Post Graduate Institute of Medical Sciences, Rohtak, IND; 2 Orthopaedics, Pandit Bhagwat Dayal Sharma Post Graduate Institute of Medical Sciences, Rohtak, IND; 3 Microbiology, PDM Dental College and Research Institute, Bahadurgarh, IND

**Keywords:** bradycardia, hypotension, caesarean section, ondansetron, spinal anaesthesia

## Abstract

Introduction

Spinal anaesthesia is frequently associated with adverse effects like maternal hypotension and bradycardia. This effect is due to a decrease in systemic vascular resistance, a decrease in central venous pressure or Bezold-Jarisch Reflex (mediated by 5-HT3 receptors). We aimed to measure the effect of three different doses of prophylactic intravenous ondansetron (5-HT3 antagonists) with a placebo on maternal haemodynamics.

Methods

A prospective randomised control study was done over 240 parturients, aged 19-35 years. They were randomly allocated into four groups (n=60) Group O4, Group O6, Group O8 and Group S to receive either intravenous ondansetron 4 mg, 6 mg, 8 mg or 0.9% normal saline respectively. Haemodynamic variables (systolic blood pressure [SBP], diastolic blood pressure [DBP], mean arterial pressure [MAP], heart rate [HR]) were recorded at 2-minute intervals for the first 20 minutes and at 5-minute intervals for further 30 minutes.

Results

A significant decrease in haemodynamic parameters was observed in group S when compared with ondansetron groups at various time intervals (p<0.05). The difference was most significant in groups O6 and O8. Development of nausea and vomiting was significantly higher in Group S compared to ondansetron groups (p< 0.005). The requirement for ephedrine was more in Group S in comparison to ondansetron groups (p<0.0001).

Conclusion

All three groups of ondansetron showed a decrease in the incidence of hypotension and use of vasopressor but Group O6 and O8 were more effective in attenuating spinal-induced hypotension in parturients undergoing caesarean section.

## Introduction

Spinal anaesthesia has become the most widely used and preferred anaesthetic technique for caesarean delivery. The advantages of spinal anaesthesia are its simplicity, reliability, rapid onset, dense motor block, excellent post-operative pain control and avoidance of potential airway complications associated with general anaesthesia. It requires small doses of local anaesthetics which reduces the risks of systemic toxicity to a minimum [1].

Despite all its advantages, spinal anaesthesia is frequently associated with adverse effects such as maternal hypotension and bradycardia. This hypotension is deleterious to both mother and foetus as it can cause maternal nausea, vomiting, dizziness, unconsciousness and placental hypoperfusion leading to foetal hypoxia and acidosis [2].

The pathophysiological mechanism involved in the occurrence of hypotension is a decrease in systemic vascular resistance and central venous pressure from the sympathetic block with vasodilatation and redistribution of central blood volume to the splanchnic circulation and lower extremities. Bradycardia can occur due to relative dominance of the parasympathetic system, increased baroreceptor activity or Bezold-Jarisch Reflex (BJR) [3].

BJR originates in cardiac sensory receptors. Diminished venous return of the blood, as observed after spinal block, not only causes stimulation of cardiac mechanoreceptors but also results in activation of thrombocytes to release serotonin (5-HT) which triggers chemoreceptors in the wall of the heart. Both these receptors when stimulated, produce BJR, which causes further bradycardia, vasodilatation and hypotension [4,5].

A number of studies conducted on both non-obstetric [6,7] and obstetric [8-23] populations suggest that 5-HT may be an important factor associated with cardiovascular responses via the BJR and this effect can be blocked at the 5-HT3 receptor by 5-HT3 receptor antagonists like ondansetron.

Since ondansetron is a common perioperative antiemetic drug, we conducted a study to verify the hypothesis that blockade of type III serotonin receptors (5-HT3 receptor) by intravenous ondansetron would reduce hypotension and bradycardia induced by spinal anaesthesia. With the null hypothesis that prophylactic intravenous ondansetron has no effect on the haemodynamics of parturients undergoing caesarean section, we aimed to measure and compare the effect of three different doses of prophylactic intravenous ondansetron (4 mg, 6 mg and 8 mg) with placebo in parturients undergoing caesarean section with respect to the following: haemodynamic parameters like heart rate (HR), systolic blood pressure (SBP), diastolic blood pressure (DBP), mean arterial pressure (MAP) and oxygen saturation (SpO_2_), the requirement of ephedrine and side effects (shivering, nausea, etc.) if any. The novelty in our study is the highest sample size per group compared to any other studies in English literature along with multiple doses of ondansetron analysed in a single study.

## Materials and methods

**Figure 1:**
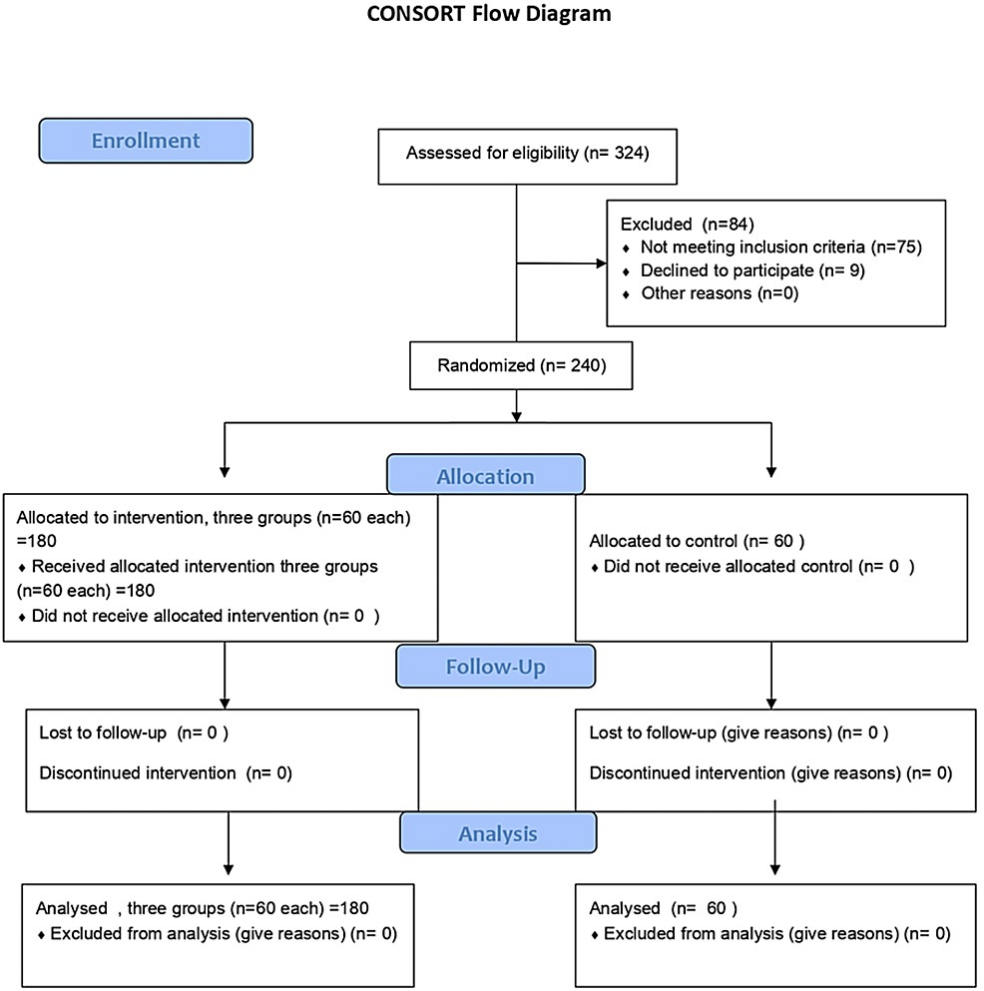
Consort flow chart

A prospective, randomised, double-blind, placebo-controlled study was conducted from 2015 to 2017 at the author’s tertiary care institute, after approval by Institutional Ethics Committee, Pt B D Sharma Post Graduate Institute of Medical Sciences, UHS, Rohtak (IEC No: IEC/Th/17/339) and was registered in the clinical trial registry, India (CTRI/2018/03/012692, Retrospectively Registered). After obtaining written informed consent, 240 term parturients in the age group of 19 to 35 years, belonging to American Society of Anesthesiologists (ASA) physical status I and II, having singleton pregnancy undergoing caesarean section with Pfannenstiel incision under spinal anaesthesia were included in the study. Patients having a history of allergy to ondansetron or local anaesthetic, pregnancy-induced hypertension (PIH) or preeclampsia, any contraindication for spinal anaesthesia, or other co-morbid conditions were excluded from the study. Our study adheres to the CONSORT guidelines (Figure 1).

All patients were subjected to complete general physical as well as systemic examination. Routine investigation according to Nice guidelines for surgery were done in all the patients including investigations for any abnormal findings. The purpose and protocol of the study were explained to the patients. Patients were kept NPO for eight hours and were premedicated with tablet ranitidine 150 mg and tablet metoclopramide 10 mg orally, two hours before the surgery with a sip of water.

Upon arrival into the operating room, all patients were laid supine with wedge under right flank to achieve leftward tilt of 15°. Routine monitoring including non-invasive blood pressure (NIBP), electrocardiogram (ECG) and oxygen saturation (SpO_2_) were established. Peripheral venous access was secured with 18-gauge cannula. All patients were pre-loaded with 10 mL/kg of ringer lactate solution over 10 minutes before spinal anaesthesia. Baseline maternal blood pressure (SBP, DBP and MAP), HR and SPO_2_ were recorded.

Patients were randomly allocated to one of the four groups of 60 patients (allocation ration 1:1:1:1) each using computerised generated randomisation technique. Opaque, sealed and sequential numbered envelopes containing group assignments were used. Group O4, Group O6, Group O8 and Group S received intravenous ondansetron 4 mg, 6 mg, 8 mg and 0.9% normal saline, respectively, with each group having equal number of patients (n=60). An anaesthesiologist not involved in patient's management and data collection verified the allocation and prepared appropriate doses of ondansetron (4, 6 or 8 mg) with 0.9% saline to a total volume of 10 ml or a placebo of 0.9% saline 10 ml in similar syringes marked "study drug''. The study drug was injected intravenously over 60 seconds, 5 minutes before lumbar puncture.

All patients were given spinal anaesthesia in sitting position. After cleaning and draping of lower back, L3-L4 interspace was identified by palpation and 25-gauge Quincke's spinal needle was inserted in the vertebral interspace. After free flow of cerebrospinal fluid 2 mL of 0.5% hyperbaric bupivacaine was administered without barbotage. Patients were immediately placed in supine position with 15° left tilt. Oxygen was given to all patients via facemask at a rate of 4 L/min. Intravenous ringer lactate was infused at constant a rate of 15 mL/kg/hr till the end of the surgery.

 Sensory block height level was checked by assessing the perception of coldness using a spirit swab at 5 minutes interval and surgery was commenced as soon as T4 dermatome was anaesthetised. Peak block height was assessed till 20 minutes. At the same time points, the level of motor blockade was assessed by Modified Bromage Scale [12]. After delivery of the foetus and clamping of umbilical cord, oxytocin infusion was started (20 units of oxytocin in 500 mL of 0.9% normal saline at 250 mL/hr, i.e., 0.16 U/mL).

Hypotension was defined as a fall in SBP of more than 20% of the baseline, was treated with boluses of 6 mg intravenous ephedrine. Bradycardia was defined as HR < 50 bpm and was treated with boluses of 0.6 mg intravenous atropine.

A second anaesthesiologist blinded to the group allocation recorded demographic data, obstetric data (indication for caesarean section, number of previous pregnancy, caesarean deliveries, uterine pathology), intraoperative time (time from dural puncture to skin incision, time from skin incision to delivery, total time of the surgery), haemodynamic variables (SBP, DBP, MAP, HR, SpO_^2^_), adverse effects (nausea, vomiting and shivering), need for atropine, ephedrine, initial and final haemoglobin values and intraoperative blood loss (measured by estimating blood collected by suction and by calculating the weight of blood on surgical swabs). Haemodynamic variables were recorded at 2-minute intervals after spinal anaesthesia for the first 20 minutes and at 5-minute intervals for further 30 minutes as well as at the end of the surgery.

Patients in whom sensory block height failed to reach T4 dermatome were excluded from the study and analgesic supplementation and/or general anaesthesia was administered as per the attending anaesthesiologist’s discretion.

Statistical analysis

With reference to previous study [9], a power analysis indicated that 60 subjects were required per group to show that the number of patients with hypotension will be 50% lower in the subjects receiving ondansetron with 80% power and at alpha 0.05 in comparison with the control group. We choose a 50% baseline ratio of number of patients with hypotension in women undergoing elective caesarean section in control group.

Statistical testing was conducted with the statistical package for the social science system version SPSS 17.0. Continuous variables were presented as mean±SD or median (Inter-Quartile-Range) for non-normally distributed data. Categorical variables were expressed as frequencies and percentages. The comparison of normally distributed continuous variables between the groups was performed using ANOVA with appropriate post hoc test. Nominal categorical data between the groups was compared using Chi-squared test. Non-normal distribution continuous variables were compared using Kruskal-Wallis test and further paired comparisons was done using Mann Whitney U test. For all statistical tests, a p-value less than 0.05 was taken to indicate a significant difference.

## Results

A total of 240 patients scheduled for elective caesarean section were recruited into four study groups (n=60) (Group O4, Group O6, Group O8 and Group S). The groups were comparable with respect to obstetric data (gestational age, previous pregnancies, caesarean section) and demographics (age, height, weight, BMI) (Table 1).

**Table 1:** Demographic profile of all the groups

	Group 1 (n=60)	Group 2 (n=60)	Group 3 (n=60)	Group 4 (n=60)	P-value
	Mean ± SD	Mean ± SD	Mean ± SD	Mean ± SD	
Age (years)	24.02 ± 2.63	24.82 ± 3.14	24.38 ± 3.59	23.82 ± 3.68	0.545
Height (m)	1.53 ± 0.05	1.53 ± 0.04	1.55 ± 0.05	1.55 ± 0.04	0.055
Weight (kg)	55.20 ± 9.88	58.18 ± 9.57	59.60 ± 9.93	61.08 ± 10.37	0.058
Body Mass Index (kg m^-2^)	23.56 ± 3.30	24.31 ± 3.49	24.80 ± 3.22	25.10 ± 3.88	0.075

**Figure 2:**
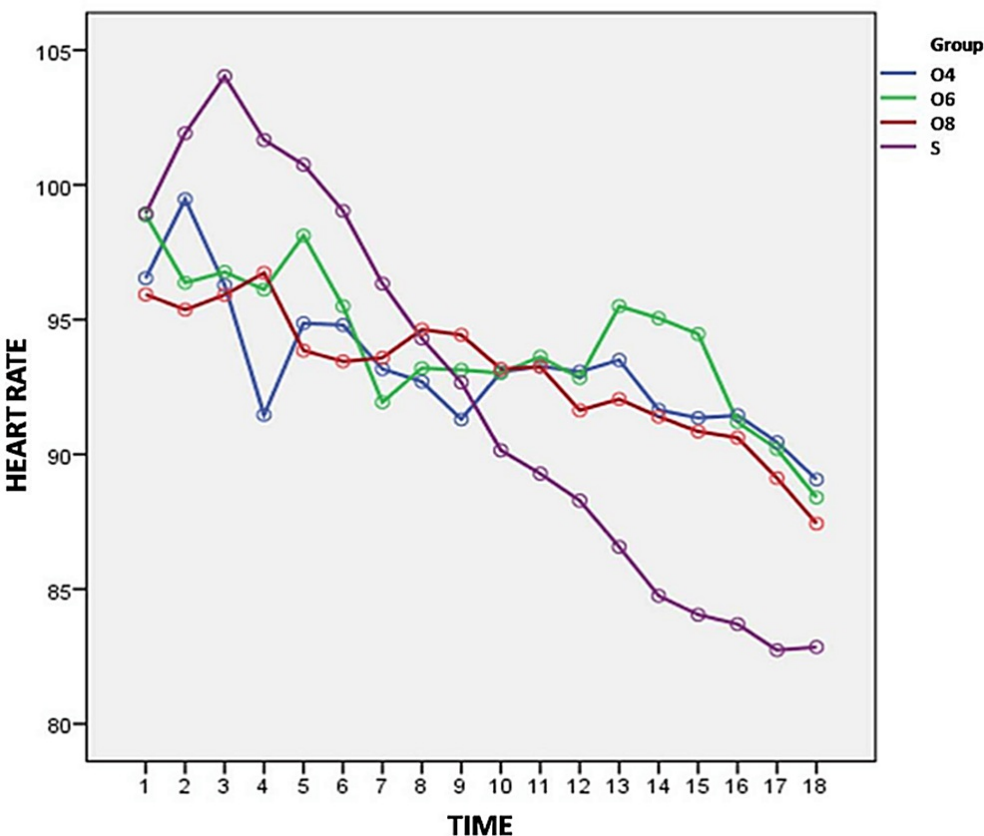
Variation of heart rate from baseline between the groups

The statistical evaluation of maternal heart rate (HR) between the four groups (Figure 2) was significant from 2 min to 8 min, and 30 min till the end of the surgery (p<0.05).

When compared with group S the mean heart rate at various time intervals were statistically significant with group O4 at 4 min (p=0.011), 6 min (p=0.001) and from 30 min till end of the surgery (p<0.05), Group O6 at 4 min (p=0.020) and from 30 min till the end of surgery (p<0.05) and Group O8 at 2 min (p=0.035), 4 min (0.006), 8 min (p=0.035) and from 35 min till end of the surgery (p<0.05).

**Figure 3:**
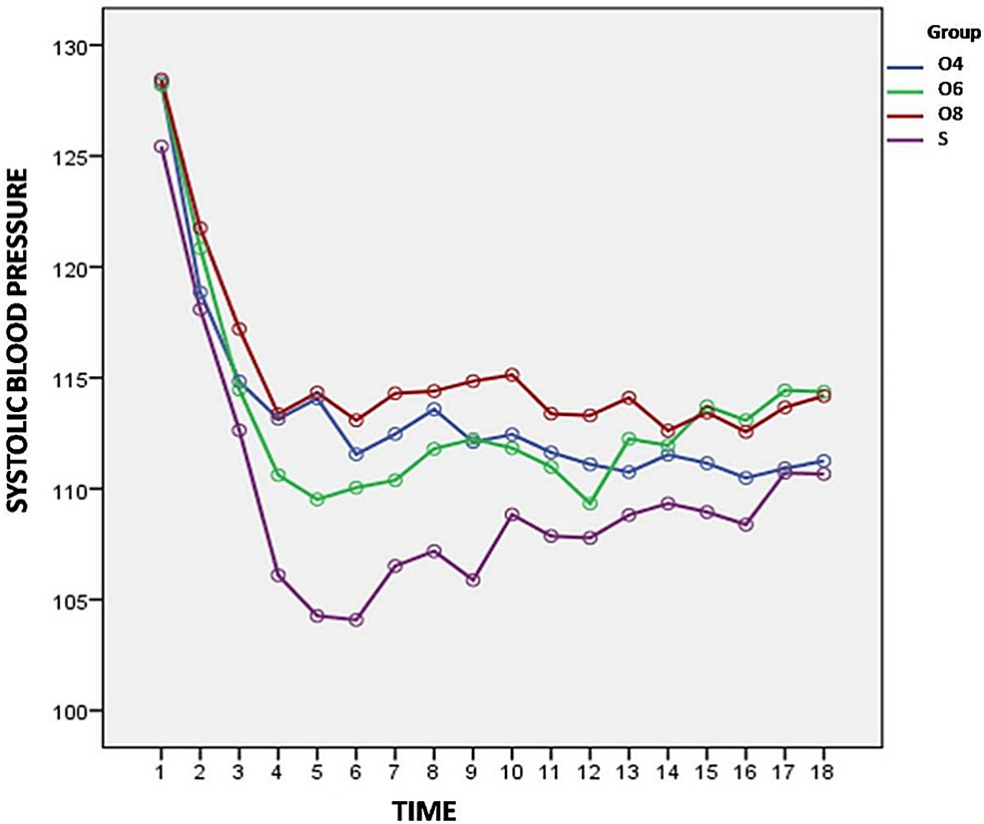
Variation of systolic blood pressure from baseline between the groups

**Figure 4:**
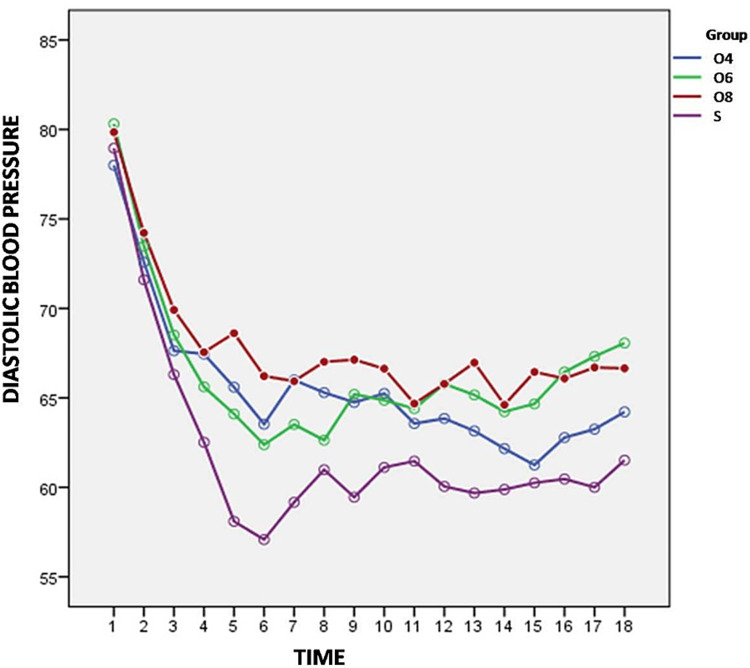
Variation of diastolic blood pressure from baseline between the groups

**Figure 5:**
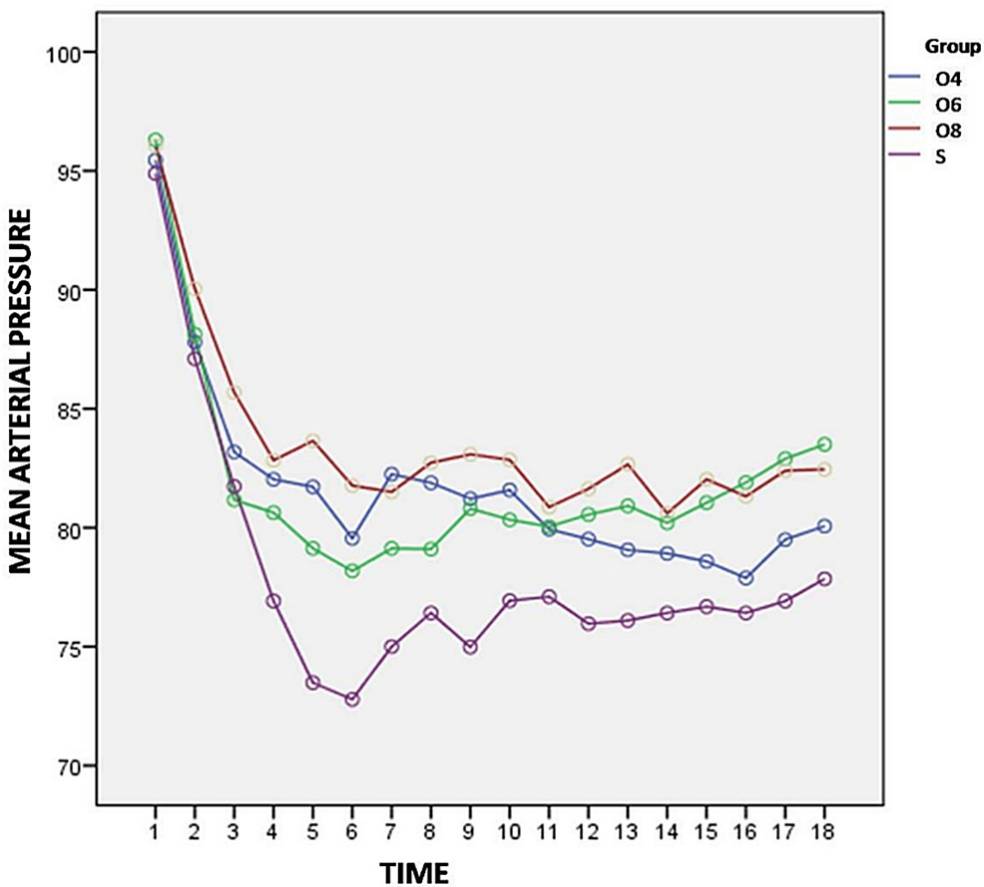
Variation of mean arterial pressure from baseline between the groups

Variation in arterial pressure (SBP, DBP, MAP) from baseline are shown in Figures 3-5.

We found significant difference in SBP recordings between the four group at all recorded time intervals except at 4 min and 35 min recordings (p>0.05). The ondansetron groups observed significantly less fall in SBP when compared with group S with statistical difference in Group O4 from 6 min to 16 min (p<0.05), Group O6 at 10 min (p=0.012), 14 min (p=0.018), 16 min (p=0.000) and from 40 min till end of the surgery (p<0.05), and group O8 at all time intervals except 4 min and 35 min (p>0.05). The number of patients with hypotensive episode (hypotension = fall in SBP > 20% of baseline) was seven (11.7%), one (1.7%), two (3.33%) and 16 (26.7%) in group O4, O6, O8 and S, respectively (p=<0.0001).

We also found statistically significant difference among the four study groups with respect to variation in DBP at all time intervals except at 2 min, 4 min, 6 min and 20 min (P>0.05). There was significant decrease in DBP in group S when compared with Group O4 at 8 min (p=0.001), 10 min (p=0.005), 12 min (p=0.002) and 16 min (p=0.006), Group O6 at 8 min (p=0.019), 10 min (p=0.037), 16 min (p=0.002) and from 25 min till end of the surgery (p<0.05) and group O8 at all time intervals except at 2 min, 4 min, 6 min and 20 min (p>0.05).

The variations in MAP were also found statistically significant among the four study groups at all time intervals except at 2 min, 4 min, and 20 min (p>0.05). We observed that fall in MAP was significantly higher in Group S when compared with Group O4 from 8 min to 18 min (p<0.05), Group O6 at 8 min (p=0.015), 10 min (p=0.016), 16 min (p=0.000) and from 25 min till end of the surgery (p<0.05) and group O8 at all time intervals except at 2 min, 4 min and 20 min (p>0.05).

Development of nausea and vomiting were significantly higher in Group S compared to ondansetron groups (O4, O6 and O8) (Table 2).

**Table 2:** Adverse reactions

Adverse reaction		Group				Total	P-value
		Group O4	Group O6	GroupO8	Group S		
Nausea	No	55	55	57	47	214	0.017
		91.7%	91.7%	95.0%	78.3%	89.2%	
	Yes	5	5	3	13	26	
		8.3%	8.3%	5.0%	21.7%	10.8%	
Vomiting	No	59	59	60	51	228	0.001
		98.3%	98.3%	100.0%	85.0%	95.0%	
	Yes	1	1	0	9	12	
		1.7%	1.7%	0.0%	15.0%	5.0%	

There was no statistically significant difference observed among groups for incidence of shivering intraoperatively (p>0.05). There was statistically significant difference (p<0.0001) in requirement of ephedrine for group S as compared to ondansetron groups (Table 3). No patient in any study group required atropine.

**Table 3:** Treatment of Hypotension

Treatment of Hypotension		Group				Total
		GroupO4	Group O6	Group O8	Group S	
Need for ephedrine	No	53	59	58	44	210
		88.3%	98.3%	96.67%	73.3%	87.5%
	Yes	7	1	2	16	30
		11.7%	1.7%	3.33%	26.7%	12.5%
Total		60	60	60	60	240
P Value		<0.0001				

## Discussion

Spinal anaesthesia is the anaesthetic technique of choice for caesarean delivery and has resulted in a reduction in maternal mortality associated with general anaesthesia. However, it is associated with maternal hypotension in 80%-83% of parturients [24]. The combined effect of reduced cardiac output and decreased systemic vascular resistance due to blockade of sympathetic nerves account for hypotension after spinal anaesthesia [25].

Ondansetron, a highly effective and specific 5-HT3 receptor antagonist, can block the binding of 5-HT to chemoreceptors, thus alleviating BJR. It also suppresses further expansion of peripheral vessels and increases venous return to the heart [23]. Although 4 mg of ondansetron preloading has been commonly used to reduce maternal hypotension and nausea, the dose-dependent effect of ondansetron on reducing maternal hypotension is still not established clearly and needs further studies [9-11,14,20-23].

Among the ondansetron groups, the mean maternal SBP, DBP and MAP when compared statistically with group S was only minimally affected in group O4 but was statistically significant at a maximal number of time points in group O6 and O8. Ephedrine consumption was also significantly less with ondansetron groups. However, a statistically significant difference in HR was observed between ondansetron groups and group S at various time intervals but no patient had any episode of bradycardia and required atropine.

Studies by Sahoo et al. [9], Trabelsi et al. [14] Wang et al. [11], and Shabana et al. [20] have demonstrated that 4 mg of ondansetron can effectively attenuate maternal hypotension during caesarean section, but all these studies only used one given dose of a drug, but our study included three different doses of ondansetron.

Ortiz et al. [13] compared two doses (2 mg and 4 mg) of ondansetron with a placebo and found that prophylactic ondansetron has little effect on the incidence of hypotension. This difference with our study may be due to the difference in the anaesethetic technique used (bupivacaine 0.06 per cm height and 20 μg fentanyl used in Ortiz et al. [13] study versus 10 mg bupivacaine in our study) and different sample size (n=32 in Ortiz et al., 13 study versus n=60 in our study).

Wang et al. [12] similar to our study compared four different doses of ondansetron (2 mg, 4 mg, 6 mg and 8 mg) with placebo and found that 4 mg and 6 mg of ondansetron are more effective in attenuating maternal hypotension than 2 mg and 8 mg. This is in contrast to our study where we found that 6 mg and 8 mg were more effective in preventing spinal-induced maternal hypotension. This difference may be due to the specific population and larger sample size of our study (n=30 each in Wang et al., 12 study versus n=60 each in our study).

Potdar et al. [26] compared two doses (4 mg and 8 mg) of ondansetron with a placebo and found that intravenous ondansetron reduced the incidence of maternal hypotension, nausea and vomiting but there was no added advantage of 8 mg ondansetron over 4 mg. This variation may be due to difference in the anaesthetic technique used (bupivacaine 12 mg and 60 μg fentanyl used in Potdar et al. [26] study versus 10 mg bupivacaine in our study).

Mohamed et al. [23] did a double-blind randomised controlled trial and compared the use of prophylactic IV ondansetron 10 mg versus normal saline in a pregnant mother undergoing elective cesarean delivery. They found no effect of ondansetron in the prevention of maternal hypotension and bradycardia. This difference from our results can be explained by the higher dose of ondansetron used in this study.

Bhiwal et al. [22] also did a study comparing two doses of ondansetron (4 mg and 8 mg) in 150 parturients and used normal saline as control. They found that prophylactic use of ondansetron was helpful in the prevention of maternal hypotension and decreases the use of vasopressor with 8 mg ondansetron having a better reduction in vasopressor requirement. This finding was similar to our study as we also found 6 mg and 8 mg as effective doses for the prevention of maternal hypotension. We have the advantage of higher sample size in our study.

Samarah et al. [21] used two different doses of ondansetron (4 mg and 6 mg) in 152 parturients and used normal saline as a control group. They administered the IV ondansetron 20 min prior to spinal anaesthesia. They found no reduction in the incidence of maternal hypotension with any of the doses of ondansetron. The different results from our study can be justified by the fact that we used prophylactic IV ondansetron 5 min prior to spinal anaesthesia.

There are a few limitations of our study. We have not compared ondansetron with any vasoconstrictors which are used for the prevention and treatment of hypotension due to spinal blockade. We have not compared ondansetron with other 5-HT3 receptor antagonists in attenuating spinal-induced hypotension and bradycardia in parturients undergoing caesarean section. But our strengths are that we have done a prospective randomised placebo-controlled, double-blinded study with the highest sample size per group compared to any other studies in English literature along with multiple doses of ondansetron analysed in a single study, and our results are well supported statistically.

## Conclusions

Spinal anaesthesia-induced maternal hypotension is a serious concern in the surgical management of pregnancy. Considering the effect on maternal haemodynamics, all three groups of patients treated with ondansetron showed a decrease in the incidence of hypotension and the use of vasopressors. However, Groups O6 and O8 were more effective in attenuating spinal-induced hypotension in parturients undergoing caesarean section.
